# PET/MR: improvement of the UTE μ-maps using modified MLAA

**DOI:** 10.1186/2197-7364-2-S1-A58

**Published:** 2015-05-18

**Authors:** Didier Benoit, Claes Ladefoged, Ahmadreza Rezaei, Sune Keller, Flemming Andersen, Liselotte Hojgaard, Adam Espe Hansen, Soren Holm, Johan Nuyts

**Affiliations:** Rigshospitalet, University of Copenhagen, Denmark; Department of Clinical Physiology, Nuclear Medicine and PET, Rigshospitalet Copenhagen, Denmark; University of Leuven, Belgium

For a quantitative analysis in positron emission tomography (PET) or single-photon emission computed tomography (SPECT), attenuation correction (AC) is mandatory. CTscans or transmission scans are common tools for determination of the attenuation μ-map, but in the case of a PET/MR hybrid system it is difficult to associate one of these scans. Many techniques have been developed in order to improve AC for PET/MR. Some methods are based on template- or atlas techniques, other methods apply a segmentation technique based on Dixon or UTE (Ultrashort Echo Time) MR to create the μ-map, followed by a standard OSEM reconstruction (OSEM/DIXON and OSEM/UTE). A different approach for AC has been developed by employing the emission sinogram data in the μ-map derivation. In this context, we modified the iterative MLAA (Maximum-Likelihood reconstruction of Attenuation and Activity) algorithm to improve the resulting emission image from the PET/MR system. We constrained the attenuation map update using the UTE μ-map and the T1-weighted (T1w) MR image in order to improve convergence towards a solution. Results show that the modified MLAA algorithm improved the estimated emission image compared to standard OSEM/UTE and OSEM/DIXON. In certain regions of the brain, in particular close to the skull and the air cavities, the modified MLAA algorithm generated less error than OSEM/UTE and OSEM/Dixon. The modified MLAA algorithm is able to compute an attenuation μ-map that is slightly more similar to the aligned CT μ-map than the UTE μ-map.

